# A FAMILY WITH LEUKONYCHIA TOTALIS

**DOI:** 10.4103/0019-5154.60365

**Published:** 2010

**Authors:** Kamran Balighi, Fatemeh Moeineddin, Vahideh Lajevardi, Rajaee Ahmadreza

**Affiliations:** *From the Departement of Dermatology, Razi hospital, Tehran University of Medical Sciences, Tehran, Iran.*

**Keywords:** *Bart pumphrey syndromes*, *leukonychia totalis*, *sensorineural hearing loss*, *palmoplantar keratoderma*, *knuckle pads*

## Abstract

A family presented to our dermatology clinic with a complaint of white nails. Physical examination revealed clinical feature of leukonychia totalis and the presence of sensorineural hearing loss, palmo plantar keratoderma and knuckle pads (four essential criteria for the diagnosis of Bart Pumphrey syndrome).Three consecutive generations of this family were affected with variable presentations of Bart Pumphrey syndrome in male and female; and autosomal dominant pattern of inheritance.

## Introduction

Bart Pumphrey syndrome (MIM: 149200) is a rare autosomal dominant syndrome, which is clinically characterized by palmo plantar keratoderma (PPK), sensorineural hearing loss (SNHL), hereditary leukonychia and knuckle pads. The underlying genetic defect of this syndrome seems to be located in the locus of connexin 26 coding gene 13q11-12 (1). Leukonychia as a sole congenital anomaly may also originate from a gene defect in the locus of 12q13, which codes a hard keratin molecule. Bart Pumphrey syndrome (BPS) is known to be autosomal dominant and no gender preferences are expected in clinical manifestations.

## Case Report

**Figure 1:**
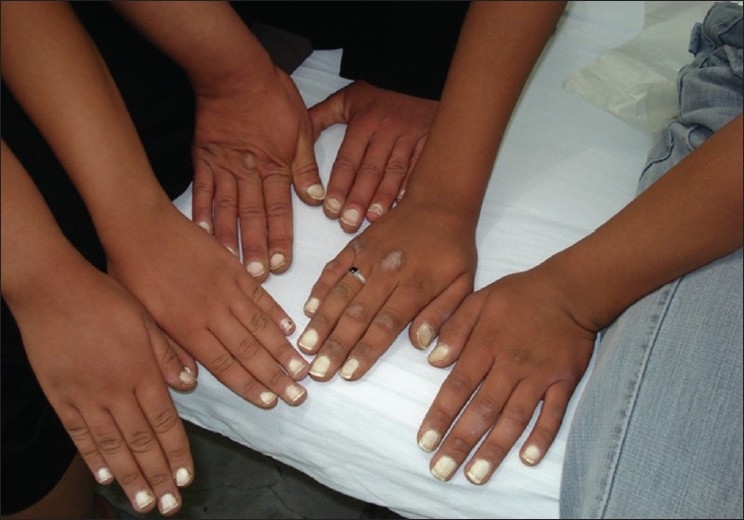
A family with leukonychia totalis

**Figure 2:**
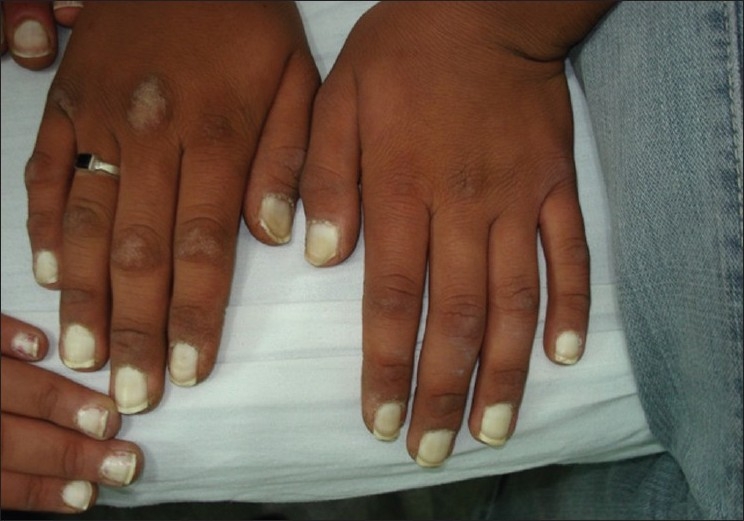
Leukonychia totalis with knuckle pads

**Figure 3:**
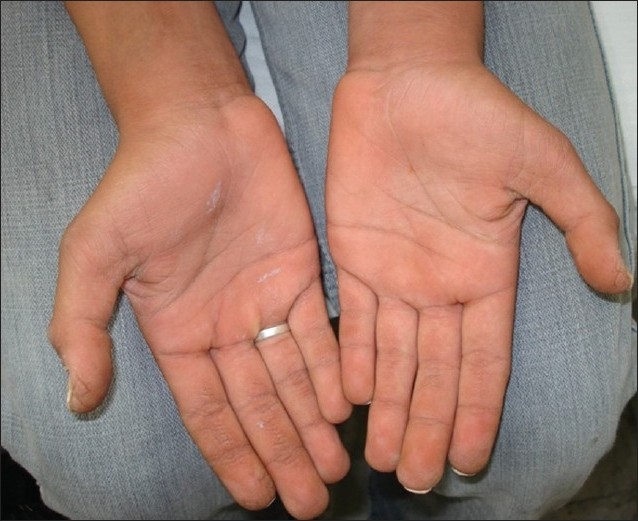
Palmoplantar keratoderma

**Figure 4:**
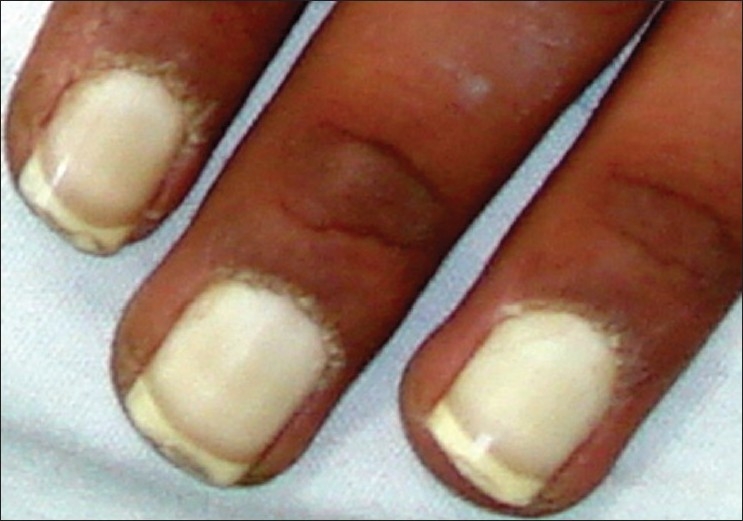
Leukonychia involving all parts of finger nails

**Figure 5:**
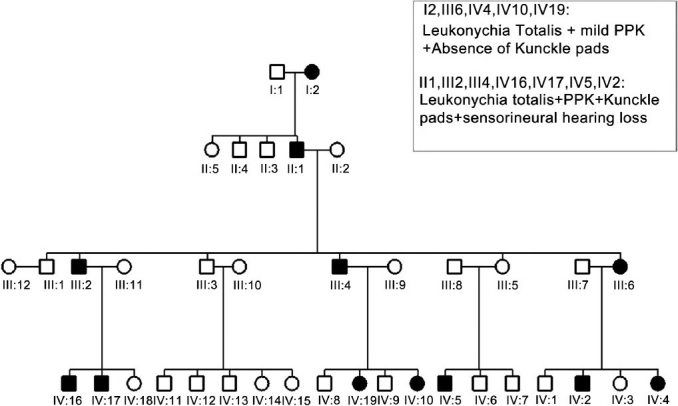
Pedigree of family with different clinical manifestations in affected males and females

We report three consecutive generations of a family presenting with leukonychia totalis [Figures 1–4]. The disorder was found in three members of a six-member family, which referred to our dermatology clinic on first visit. Leukonychia totalis, palmoplantar keratoderma, SNHL and knuckle pads were present in variable severity in the three mentioned cases as well as in the maternal uncle, aunt and grandparents, as shown in the pedigree [Figure 5]. Clinical findings of the described cases were compatible with the autosomal dominant pattern of inheritance with variable manifestations in males and females.

## Discussion

There are several nail disorders leading to white discoloration; hereditary leukonychia is an infrequent cause. Leukonychia may either involve all regions or just a limited area of a nail; known as total and partial hereditary leukonychia respectively. Since both entities may be found separately in some persons at different times, it seems that the two conditions are probably variable presentations of a single disorder.[1]

Leukonychia totalis may present as a sole congenital anomaly or in association with many other dermatologic problems. Some of the associated abnormalities reported so far include; palmoplantar keratoderma, pilar and sebaceous cysts, severe keratosis pilaris; pili torti, hypotrichosis; onychorrhexis and koilonychia. These overlapped findings suggest a possible single cause, most probably related to the mutation of a gene, which regulates the structure of a hard keratin.[2–4] Genetic evaluation on four generations of a French family with hereditary leukonychia as a sole congenital anomaly and autosomal dominant pattern of inheritance provided evidence of linkage to chromosome 12q13, a region which contains the basic type II keratins locus.[5]

However, leukonychia can also present as part of a distinct syndrome. About four decades ago, in 1967, Bart and Pamphrey described a new autosomal dominant syndrome characterized by palmoplantar keratoderma, leukonychia, knuckle pads and deafness in a six-generation family and suggested the presence of a single gene defect causing all those phenotypic presentations. SNHL can also present in association with some known other inherited dermatologic syndromes; the most common of which include:

1. Vohwinkel syndrome (MIM: 124500): An autosomal dominant disorder characterized by congenital SNHL, palmoplantar keratoderma and bandlike constrictions of fingers and toes that may cause auto amputation.
2. KID syndrome (MIM: 148210): An autosomal dominant entity involving SNHL, palmoplantar keratoderma, erythrokeratoderma, keratitis, corneal neovascularization hypotrichosis and atrichosis. Abnormalities of nails include partial leukonychia; and knuckle pads.
3. PPK/deafness syndrome: Was defined with diffuse palmoplantar hyperkeratosis in association with late onset SNHL, again with autosomal dominant pattern of inheritance, although some reports of mitochondrial point mutation leading to maternally inherited syndrome also exists.

The constant presence of SNHL and close overlap in associated dermatologic abnormalities also suggests a single genetic etiology.

Gabriel Richard *et al*. show that BPS results from a point mutation in the GJBZ gene on chromosome 13q11-12, which codes the protein connexin-26; this gene is responsible for two other overlapping syndromes; Vohwinkle and KIDS.[6]

This finding would support Bart and Pumphrey's hypothesis of a single genetic defect as the distinct etiology. BPS shows considerable phenotypic variability.

Bart and Pumphrey stated the presence of SNHL in all reported family members. PPK and leukonychia and knuckle pads were noted in lower prevalence.[7] Some features may present in an age-dependent pattern; palmoplantar keratoderma, defined as diffuse sharply demarcated thickening of the skin of palms and soles, usually exacerbate as the patient reaches the adolescence period.[7–9]

Ramer *et al*., reported the case of five members of a family who presented with variable manifestations of BPS without leukonychia totalis; SNHL was noted as a constant finding in all of them.[8]

In another report, a 22-year-old man presented with knuckle pads, SNHL, PPK, and absence of leukonychia similar to Remer findings in the previous report. Autosomal dominant pattern of inheritance with male to male transmission is another finding which has been suggested in this report.[9]

As mentioned, no gender preference was noted in the manifestation of BPS in several previous reports. However, in the family presented here, there were noted striking and considerable gender preference in some manifestations.

Leukonychia totalis was present as the most common manifestation in all affected members while knuckle pads were only present in male members.

SNHL was not detected in any female member of these three generations, while all male cases were affected from early childhood.

Pilar keratosis was also present in III6 and IV4 [Figure 5] on buttocks and arms, but none of the other members were affected. Palmoplantar keratoderma, in various severities, was present in almost all cases with the manner of decreasing severity from males to females. PPK had been exacerbated with age.

## Conclusion

Review of presented pedigree approves the autosomal dominant pattern of inheritance for BPS, but suggests an association between disease presentations and patient's gender, unlike previous literature. The clinical manifestations in two genders may be just an accidental finding or the result of specific etiologic factors.
